# Supplementary material for: Fully automatic transfer and measurement system for structural superlubric materials
Source: Nat Commun. 2023 Oct 10;14:6323. doi: 10.1038/s41467-023-41859-6 (PMC10564961; doi:10.1038/s41467-023-41859-6)
Supplement: Supplementary file 1 — Supplementary Information [file 41467_2023_41859_MOESM1_ESM.pdf]

Supplementary information for

Fully automatic transfer assembly system for micron materials/Devices

Li Chen[1,2], Cong Lin[3], Diwei Shi[1,2], Xuanyu Huang[2,6], Quanshui Zheng[1,2,4,5], Jinhui Nie[4*] & Ming Ma[2,4,6*]

[1] Department of Engineering Mechanics, Tsinghua University, Beijing 100084, China

[2] Center for Nano and Micro Mechanics, Tsinghua University, Beijing 100084, China

[3] Department of Computer Science and Engineering, University of California, San Diego, CA 92093, U.S.A.

[4] Research Institute of Tsinghua University in Shenzhen, Shenzhen 518057, China

[5] Institute of Materials Research, Shenzhen International Graduate School, Tsinghua University, Shenzhen 518055, P. R. China.

[6] Department of Mechanical Engineering, Tsinghua University, Beijing 100084, China

Corresponding authors: maming16@tsinghua.edu.cn, niejh@Tsinghua-sz.org

Inventory of Supplementary Information:

Supplementary Figures 1 to 12

Supplementary Notes 1 to 8

1. GUI design for transfer and measurement system.
2. The influence factors of deep learning algorithm.
3. Influences of flake and tip.
4. Automated self-retraction force measurement.
5. Time analysis for transfer graphite flakes.
6. Influence of transferred flake thickness
7. Friction measurement of transferred graphite flakes.
8. The performance of deep learning algorithms.

Supplementary Table 1

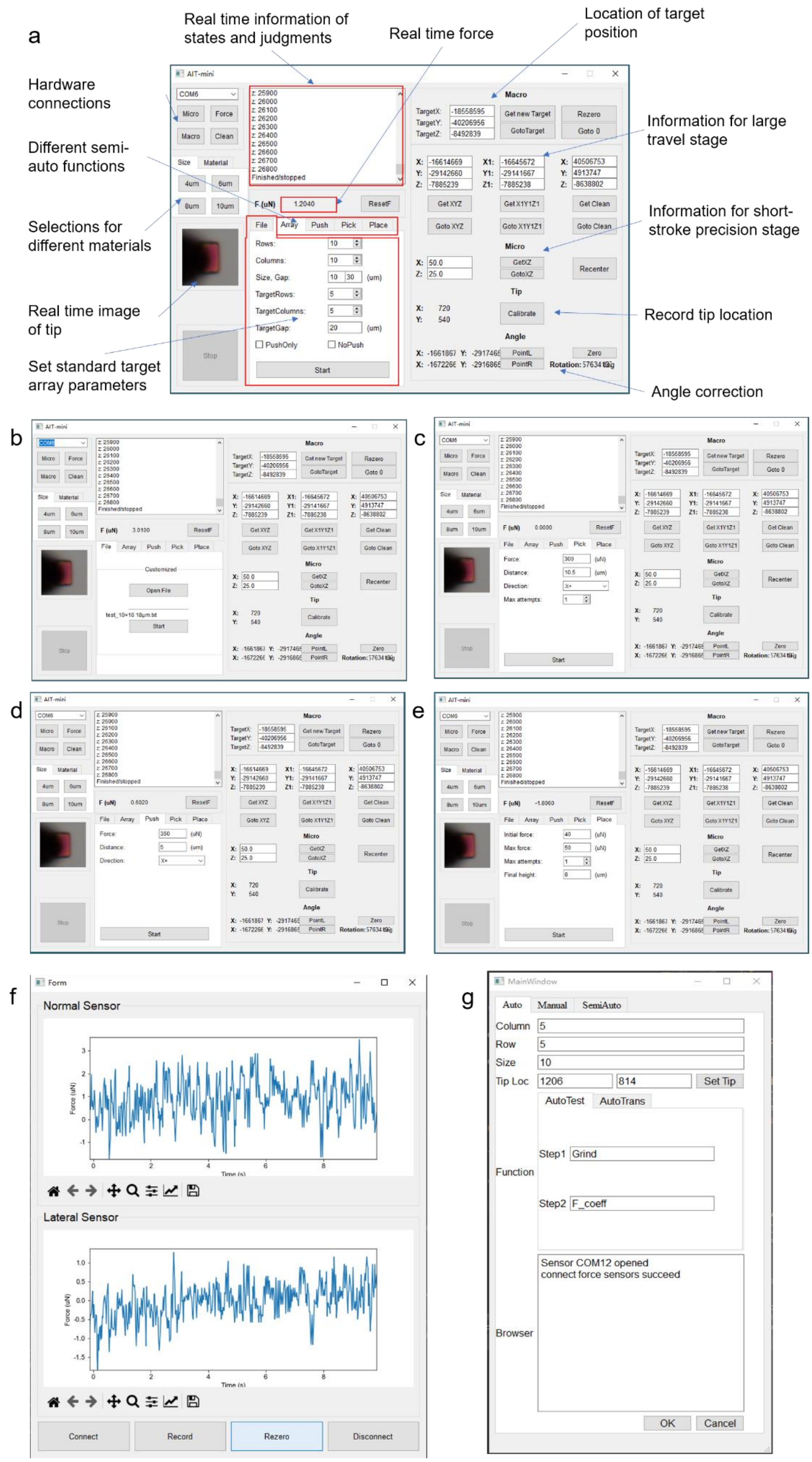

Supplementary Figure 1. Detailed graphical user interface (GUI) window screenshots of our

CAD software. **a** Fully automatic transfer interface and descriptions of the functions of each section. **b** Detailed custom target array transfer interface. **c-e** Semi-automatic functions for different transfer step: (c), picking (d), pushing (e), placement. **f** Additional form for displaying force sensor signals in real-time, where user can save signals through “Record” button. **g** GUI of automatic mechanic test, where selected functions can be operated to multiple flakes.

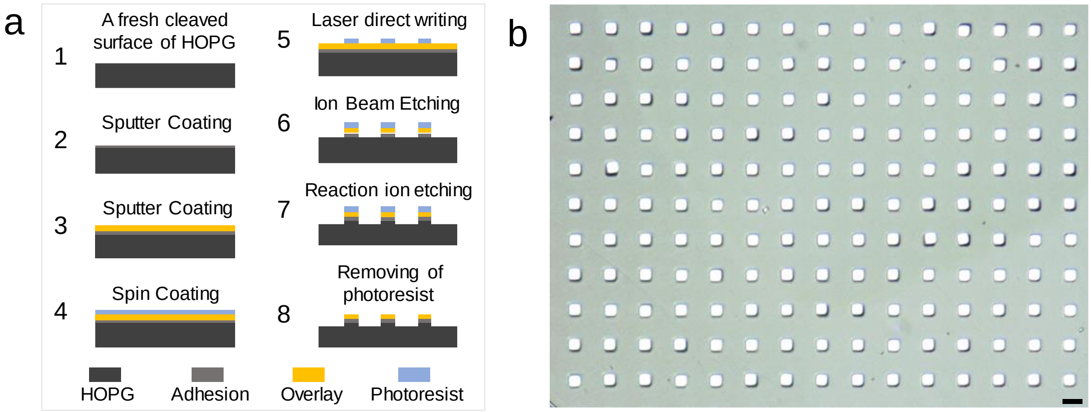

Supplementary Figure 2. Schematic and results of generating graphite flakes. **a**, The schematic of the production process for SSL graphite flakes. **b**, Microscope view of the prepared graphite flakes and the scale bar is 10 μm.

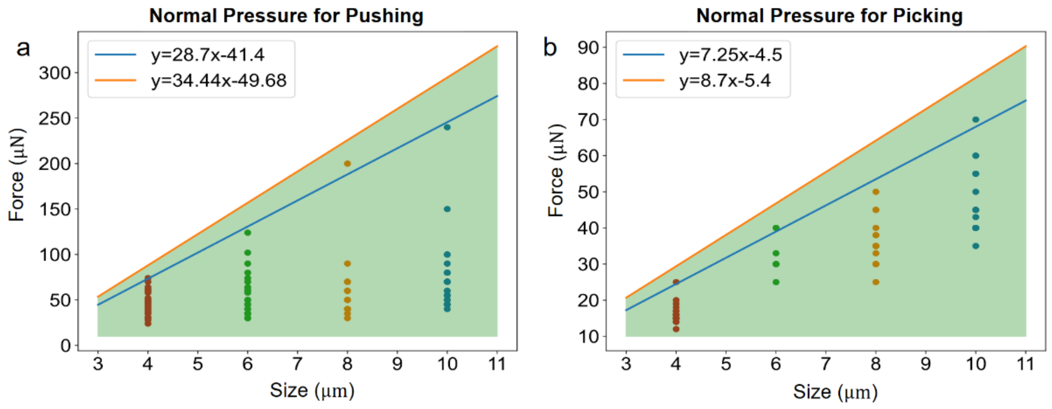

Supplementary Figure 3. Normal pressures introduced for the first time pushing (**a**) and after self-recovery picking (**b**) for different sizes of graphite flakes. The different colored dots are measurements of the normal pressure for pushing the different sizes of graphite flakes. The blue lines show the result of the linear fit to the maximum positive pressure and the orange lines show the positive pressure after retaining a 20% margin, and the final transferred force parameters are calculated from the two orange lines.

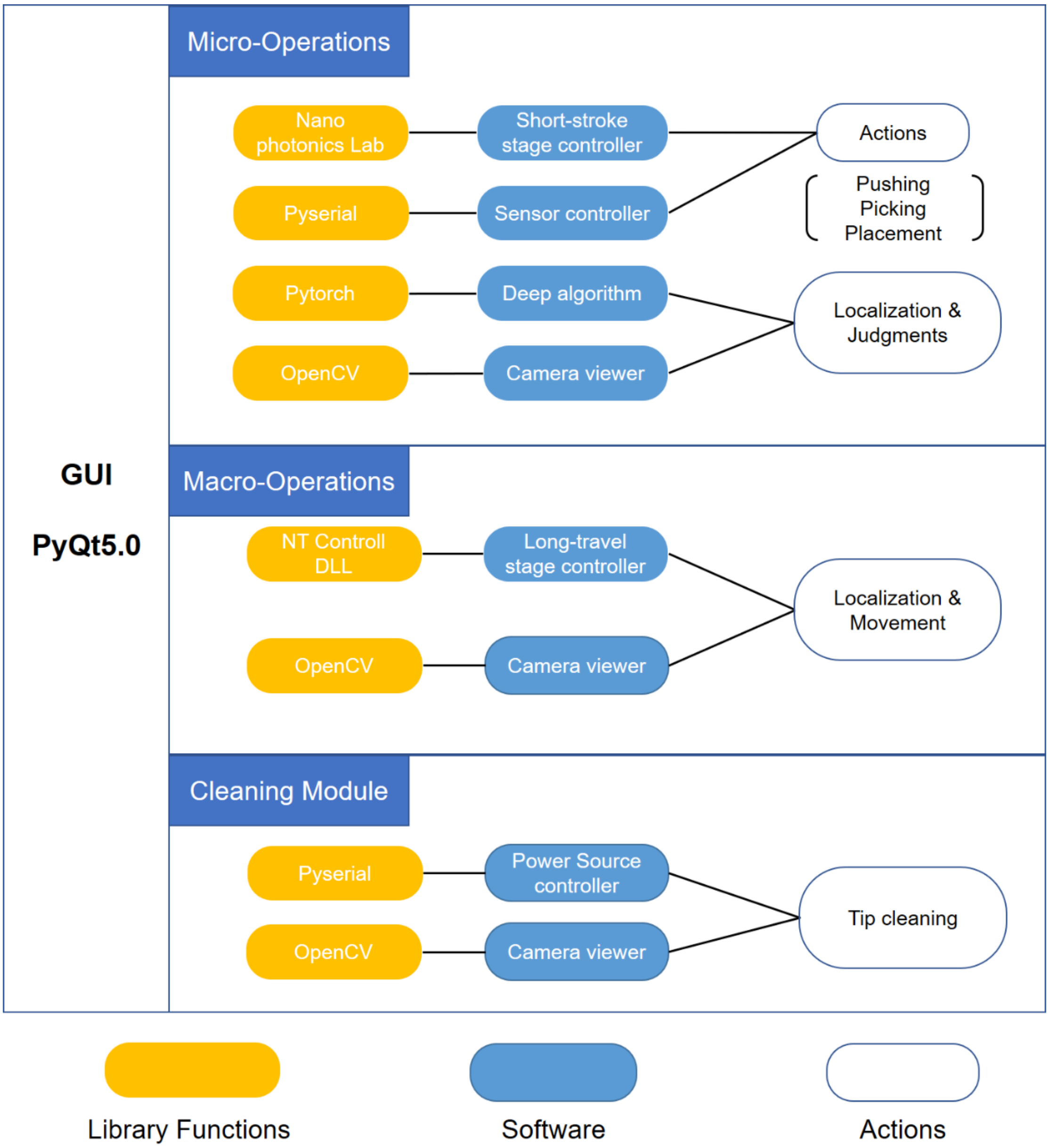

Supplementary Figure 4. The connectivity diagram of the software components in the system. The GUI consists of three major components: micro-operations, macro-operations and cleaning module. Each component sends commands through the presented libraries and python-Qt slot functions. The software and image processing algorithms are provided with different libraries; thus, the algorithms can be switched without shutting down the software.

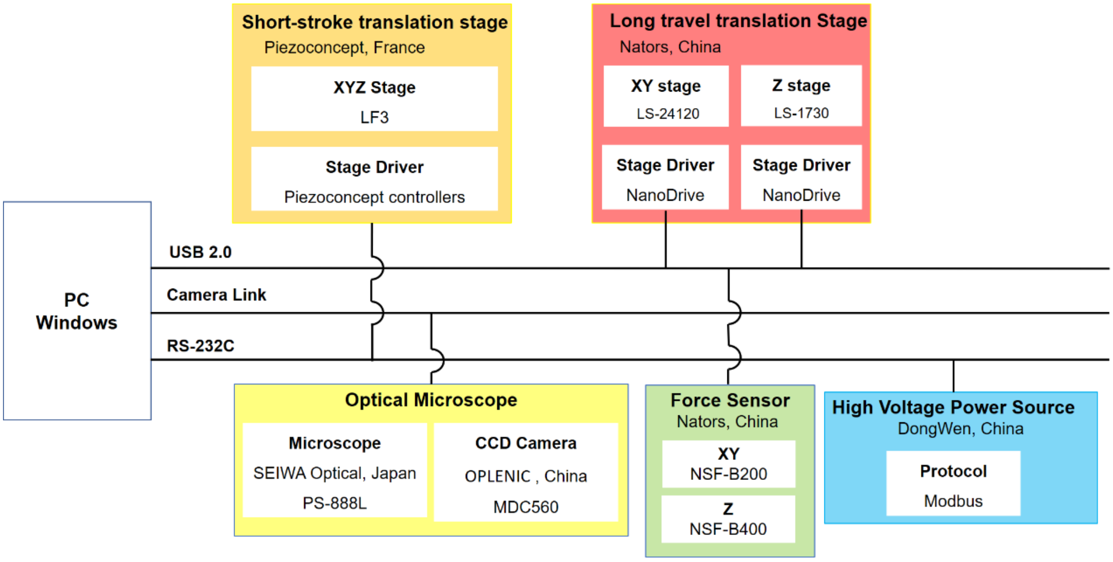

Supplementary Figure 5. The connectivity diagram of the hardware components utilized in the system.

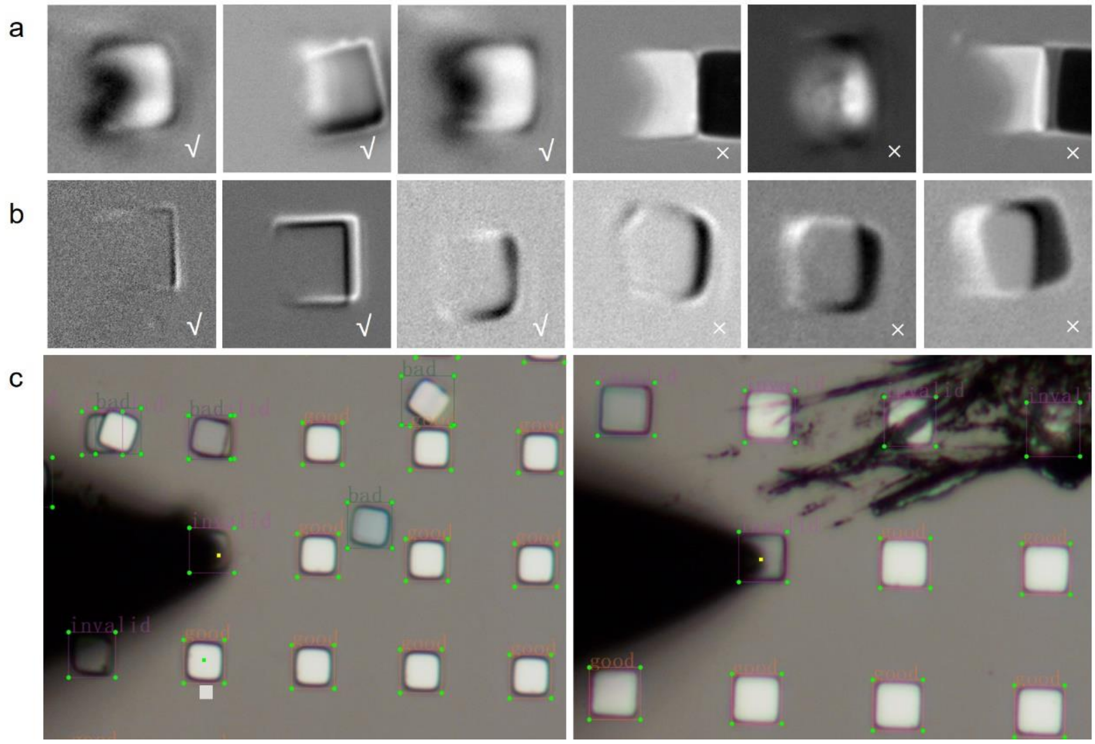

Supplementary Figure 6. Some sample data in our collected datasets, where √ means positive sample and × means negative sample. **a** Samples for determining whether a graphite flake is on the probe. **b** Samples for determining whether a graphite flake is superlubricity. **c** Samples for position target flakes and tungsten tip.

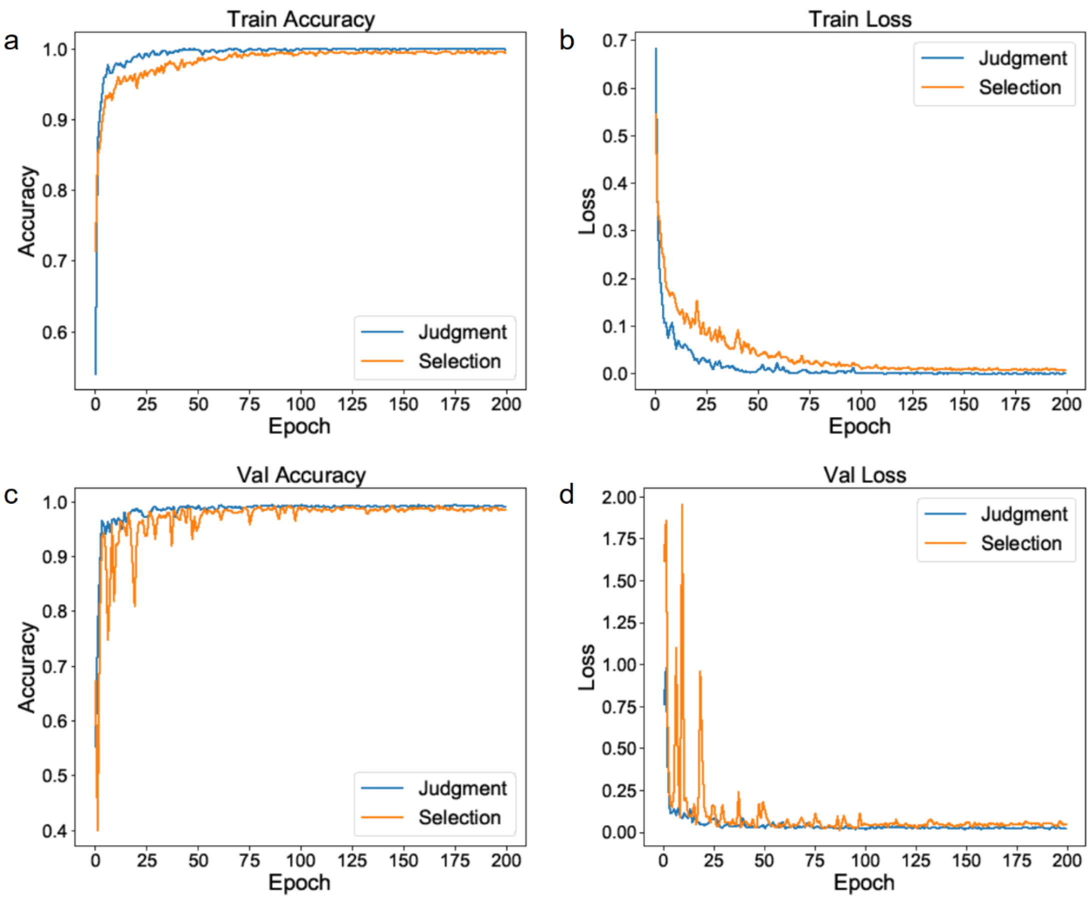

Supplementary Figure 7. Different model performance metrics during training, **a** training accuracy; **b** training loss; **c** validation accuracy; **d** validation loss. The blue line shows the model for determining whether the graphite flake is on the probe and the orange line indicates the model for determining whether the graphite flake is SSL.

Supplementary Notes

Supplementary Note 1

GUI design for transfer and measurement system. In Supplementary Figure 1, we show the GUI for each of the different functions. The main interface, as presented in Supplementary Fig. 1a, consists of three parts: 1) an information display section containing real-time information about the force sensor, the image of the tip section, the position of each displacement platform, etc. In this section, we have also added other interactive functions such as manual tip position calibration, displacement stages, force transducer zeroing and angle calibration. 2) A hardware connection section to connect to the corresponding hardware by selecting the cluster communication (COM) port. The serial number of the COM ports in this connection section are automatically searched for avoid manual checking. 3) A transfer parameter setting and semi-automatic selection interaction section for selecting the specific function required. In this section, users can transfer the SSL materials fully or semi-automatically according to the needs of the experiment or assembly. In addition to the function of automatic transfer, we have also developed a software interface for automatic mechanic testing, as shown in Supplementary Fig. 1f and 1g. The signals of normal and lateral forces are displayed in real-time in the interface, while the user can save the required signals through the “Record” button. Besides, the force signals are also emitted in real-time to the XYZ precision stage and the main program for calculating the friction force during the test.

Supplementary Note 2

The influence factors of deep learning algorithms.

1. Optical microscope objective magnification and pixel resolution: The extreme spatial resolution of our system is:

$$d = \frac{0.61\lambda}{N.A.} \approx 0.8\ \mu m \tag{S1}$$

Where N.A. indicates the Numerical Aperture, which equals 0.42 for 50× microscope in our system and λ is the average wave length of visible spectrum and equals 550 nm. The spatial resolution of the acquired image, determined by factors such as the objective magnification and pixel resolution, plays a crucial role in the deep learning algorithm. For objective magnification, Supplementary Fig. 8a shows the 8μm flakes at 10× (left), 20× (middle), and 50× (right), and the localization algorithm can identify the location of graphite flakes and select those that are not contaminated or damaged. But considering the scale of the image, 50× is utilized as the final magnification for localization. And for pixel resolution, Supplementary Fig. 8b presents the images with different pixel resolutions of 720×960 (left), 1080×1440 (middle), 1440×1920 (right). The results show that higher pixel resolution contributed to more precise flake bounding boxes.

2. Illumination condition of the microscope: Supplementary Fig. 8c showcases the localization results under varying illumination conditions. The localization algorithm demonstrates resilience to changes in lighting conditions, a feature largely attributable to the use of data augmentation methods such as luminance transformation during the training phase. This ensures that the algorithm maintains its performance and continues to deliver accurate results irrespective of the lighting conditions, thereby underlining its robustness and reliability.

a

b

c

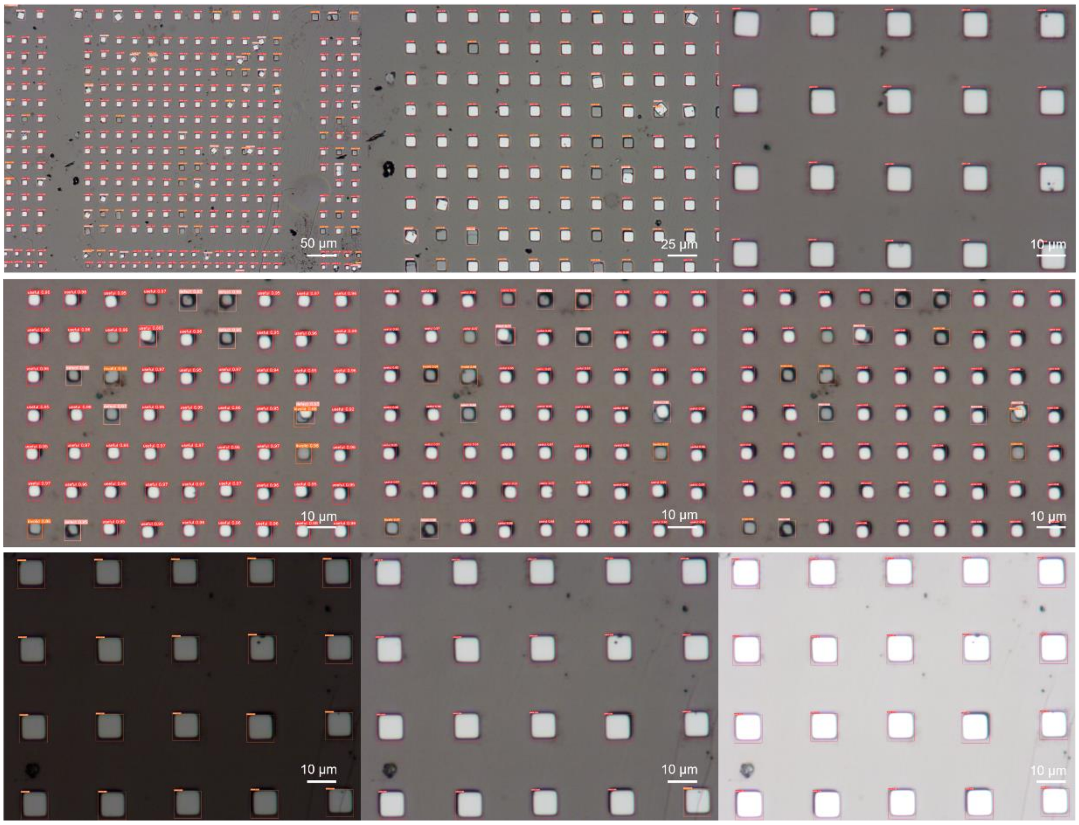

Supplementary Figure 8. The influence of optical condition to deep learning algorithm. **a** different objective magnitude. **b** different pixel resolution. **c**. different illumination condition.

Supplementary Note 3

Influences of flake and tip.

1. Size of flakes: In Figure 2, the system has shown the ability of transfer graphite flakes with 4-10 μm, which is commonly utilized and has high SSL properties. For graphite flakes smaller than 4 μm, limited by the errors of the localization algorithm, the tip may contact nearing the flake edges when operating, causing a lower transfer rate. For graphite flakes larger than 10 μm, as shown in the Supplementary Fig. 9a, the system found that the success rate of picking the graphite flakes with the size of 20 μm is low, probably due to insufficient van der Waals forces between the tip and the mesa. However, previous research indicated that the SSL probability for 20 μm graphite flakes is almost zero, causing low research value[1].

2. Size of the tip: The size of the probe tip has a relatively small impact on the transfer of graphite flakes. However, to improve the success rate of the transfer, probes with different curvature radii should be chosen for graphite flakes of different sizes. When the size of the graphite flake is smaller than 6 μm, it is preferable to use a probe with a curvature radius of 2 μm, as this facilitates precise positioning. When the graphite island is larger than 6 μm, it is preferable to choose a probe with a curvature radius of around 5 μm. This increases the van der Waals force between the graphite flake and the probe tip, thereby increasing the success rate of the transfer.

3. Position of the tip/flake contact: Supplementary Fig. 9b show how to calibrate the tip position. First, the tip is pressed onto a highly oriented pyrolytic graphite (HOPG), creating a depression (left). Then, the tip is lifted and moved 10 μm to the left. The depression location is recorded as the actual contact position and used to train the tip positioning algorithm. Therefore, the predicted tip position corresponds to the contact position between the tip and the flake mesa. During the transfer process, the system moves the tip to the center of the graphite flake. Considering a positioning error of 1 μm, the contact position of the tip/flake contact have a range of 1μm around the center of the flake. Due to the final transfer success rate of over 94%, the 1 μm error has little

effect on the transfer success rate.

a

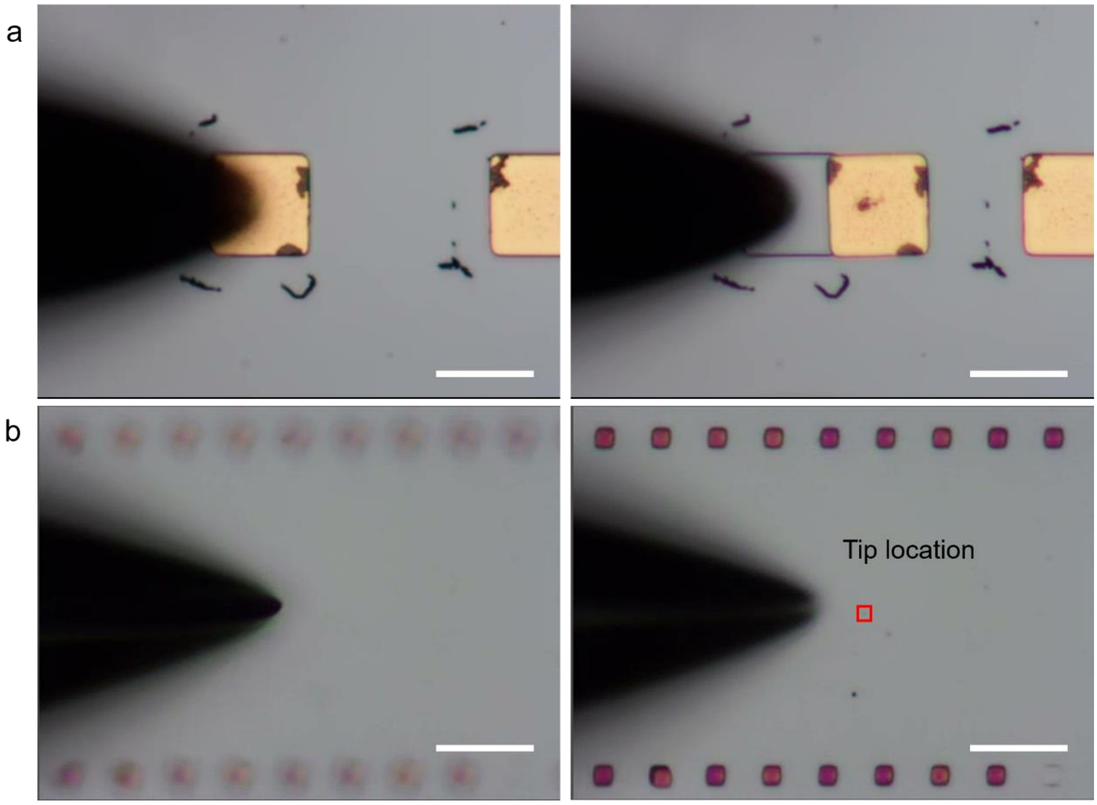

Supplementary Figure 9. The influence of flake size and tip location to the transfer process. **a** picking result for 20 μm graphite flake. **b** illustration of calibrating the tip position.

Supplementary Note 4

Automated self-retraction force measurement. Besides the transfer process, automatic measurement is another intrinsic ingredient for the upcoming SSL industry as it provides us the ability of auto-selection of the optimal materials for achieving SSL under certain working conditions. To verify the automatic measurement capability of our system, we automated the operations in measuring the basal plane cleavage energy (CE)[2] Γ of graphite, which is a key material parameter for graphite, graphene, and carbon nanotubes. Specifically, in the superlubric state, the dissipation can be neglected, and there exists a tendency for the flake to retract back to its original position to reduce the free energy. The total free energy changes G by shearing a flake of size B with a distance of x satisfies that $G = \Gamma Bx$, which introduces a driving force, $F_{\mathrm{ret}} = (dG)/(dx) = \Gamma B$. Therefore, the cleavage energy Γ can be determined through precise measurement of the applied shear force, F_{app}, required to balance the retraction force F_{ret} in the quasi-static loading (shearing) and unloading (retraction) processes, i.e., $\Gamma = F_{\mathrm{app}}/B$. The final automated experimental results are presented in Supplementary Fig. 10. The results show that $\Gamma = 0.36 \pm 0.04$ J/m^2, which agrees with the reported values[2] (0.37 ± 0.01 J/m^2). Such a good agreement demonstrates the ability of our automated system in delivering accurate and reliable measurement results. The corresponding efficiency is 5 times higher than manual operation for a well-trained engineer.

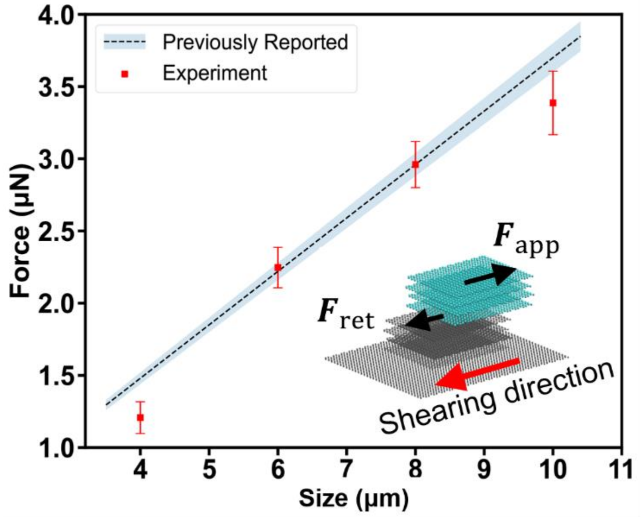

Supplementary Figure 10. The self-retraction force measurement by the automated system. The system selects the SSL flakes and shears the mesa as the inset image and then records the lateral force for calculating the retraction force. The experiment results of our automated system

compared with the previously reported ones[2], where each data point contains 25 SSL samples and error bar represents the standard deviation of the self-retraction forces for those samples.

Supplementary Note 5

Time analysis for transfer graphite flakes. The current basic manufacturing processes relies on the manual operation of the experimental apparatus. Considering the yield rate P_1, the expected time needed to transfer N micro graphite flakes with the incommensurate interface as $(A/P_1 + B/P_2 + C) \times N$, where A is the time for pushing the flakes, B is the time for picking them onto tips, C is the time for transfer to the target position, and P_2 is the success rate for picking. For the current process of generating graphite flakes, $P_1 \approx 60\%$. When we estimate $A = 2$ min, $B = 1$ min, $C = 10$ min, and $P_2 = 70\%$, which can be an optimistic value even for a well-trained researcher, the time required to complete a millimeter-scale macrostructure superlubricity which requires at least $N = 1000$ flakes with incommensurate counts up to ~250h of successive process. Performing such repetitive manual tasks is practically impossible.

Supplementary Note 6

Influence of transferred flake thickness. The thickness of the transferred flakes can vary due to the height differences of the SSL interfaces of different flakes. To accommodate for the assembly of multiple flakes with diverse heights, a bonding technology has been employed to address these variances. As shown in Supplementary Fig. 11, the thickness of the bonding material can be precisely controlled to be above 1 μm, allowing for a height tolerance of 1 μm for graphite flakes. Given that the etching depth during the preparation of graphite islands is 1 μm, the maximum height difference among the graphite flakes does not exceed this measurement. Consequently, wire bonding technology effectively addresses the issue of height variation in graphite flakes.

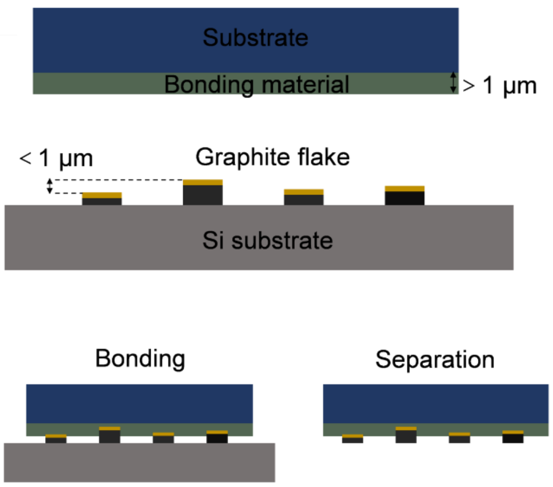

Supplementary Figure 11. Bonding process diagram, the height differences of graphite flakes are resolved by bonding material thickness.

Supplementary Note 7

Friction measurement for transferred graphite flakes. Supplementary Fig. 12 shows the friction of the graphite flakes transferred onto graphite substrates by the automated transfer system. The friction coefficients of the six groups tested range from 0.0013 to 0.0031, which are all less than 0.01, indicating that the transfer process of the system will not affect the SSL performance of the graphite flakes.

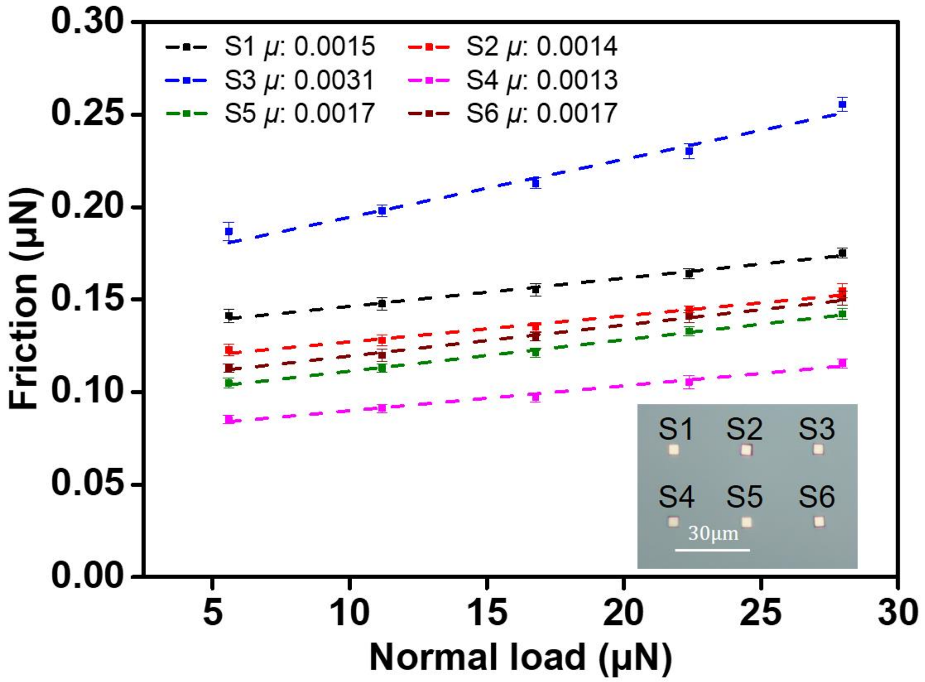

Supplementary Figure 12. Friction for the transferred graphite flakes measured under different loaded conditions by AFM, where the error bars represent the standard deviation of 25 independent friction loops, all with a coefficient of friction of less than 0.01

Supplementary Note 8

The performance of deep learning algorithms. The additional test datasets have been collected to evaluate the robustness of our trained models, where the performances for each task are presented in Supplementary Table 1. For each judgment task, our system transferred SSL flakes and collected over 1,000 input images simultaneously, and on images not trained by these models, each model performed well, with an accuracy of over 98%, and an F1 score of over 96%. Besides, it is worth noting that the size of our trained models is only 9 MB and each model takes less than 200 ms to return its determination, ensuring the efficiency of our transfer system.

Supplementary Table 1 The judgment results of this system for the three key decisions

Task	Positive	Negative	TPR①	TNR②	F1-score	Accuracy
Recover	1176	2204	98.21%	98.09%	0.977	98.14%
Place	1176	141	99.15%	99.29%	0.965	99.16%
Pick	1924	2482	99.43%	99.23%	0.993	99.34%

① TPR: True Positive Rate

② TNR: True Negative Rate

References:

1 Liu, Z. et al. Observation of microscale superlubricity in graphite. *Phys. Rev. Lett.* **108**, 205503 (2012).

2 Wang, W. et al. Measurement of the cleavage energy of graphite. *Nat. Commun.* **6**, 7853 (2015).
