# Supplementary material for: Fully automatic transfer and measurement system for structural superlubric materials
Source: Nat Commun. 2023 Oct 10;14:6323. doi: 10.1038/s41467-023-41859-6 (PMC10564961; doi:10.1038/s41467-023-41859-6)
Supplement: Supplementary file 3 — Description of Additional Supplementary Files [file 41467_2023_41859_MOESM3_ESM.pdf]

Description of Additional Supplementary Files

File Name: Supplementary Movie 1
Description: The system records the position of the target substrate, and calculates the coordinates of the target position with a set pattern.

File Name: Supplementary Movie 2
Description: The system records the position of graphite flakes and the user sets up the transfer parameters through the designed GUI.

File Name: Supplementary Movie 3
Description: The entire operation and transfer process. The force sensors, translation stages, and machine learning algorithms work jointly to search and record the nearest graphite flake, push the target flake, determine whether it has SSL property and transfer the SSL flake.

File Name: Supplementary Movie 4
Description: The entire transfer operations for assembling the pentagram array.

File Name: Supplementary Movie 5
Description: The entire tribological measurement process for graphite flakes on Si_3N_4 substrate.

File Name: Supplementary Software 1
Description: The codes of the designed software and hardware controller.
