# Supplementary figures and images for: Fully automatic transfer and measurement system for structural superlubric materials
Source: Nat Commun. 2023 Oct 10;14:6323. doi: 10.1038/s41467-023-41859-6 (PMC10564961; doi:10.1038/s41467-023-41859-6)

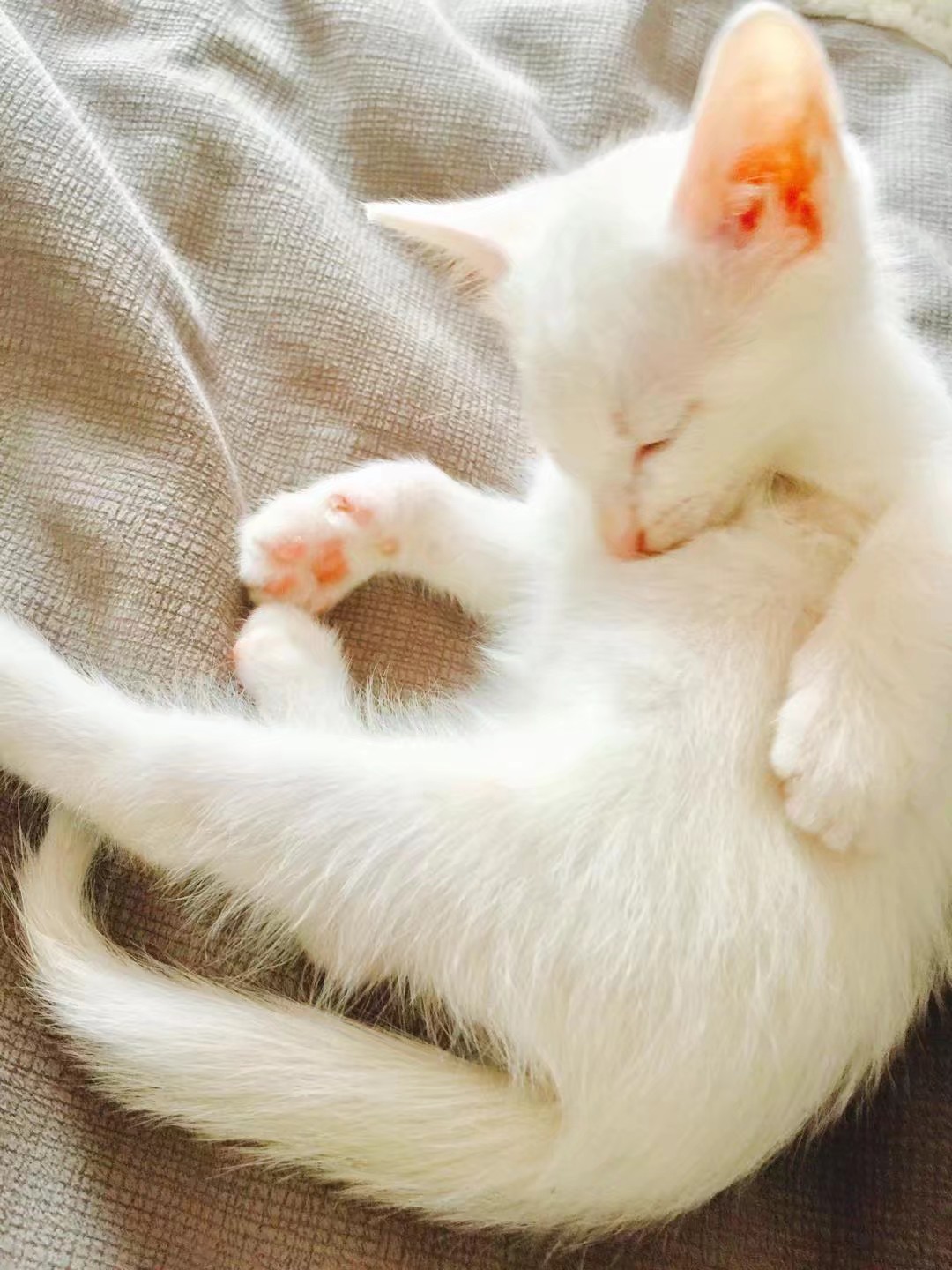

Supplement: Supplementary file 9 — Supplementary Software 1 [file 41467_2023_41859_MOESM9_ESM.zip › Supplementary Software/AutoTest/cat.jpeg]

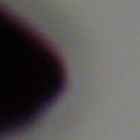

Supplement: Supplementary file 9 — Supplementary Software 1 [file 41467_2023_41859_MOESM9_ESM.zip › Supplementary Software/AutoTransfer/gui/1652839745_[0.39444444444444443, 0.65].png]

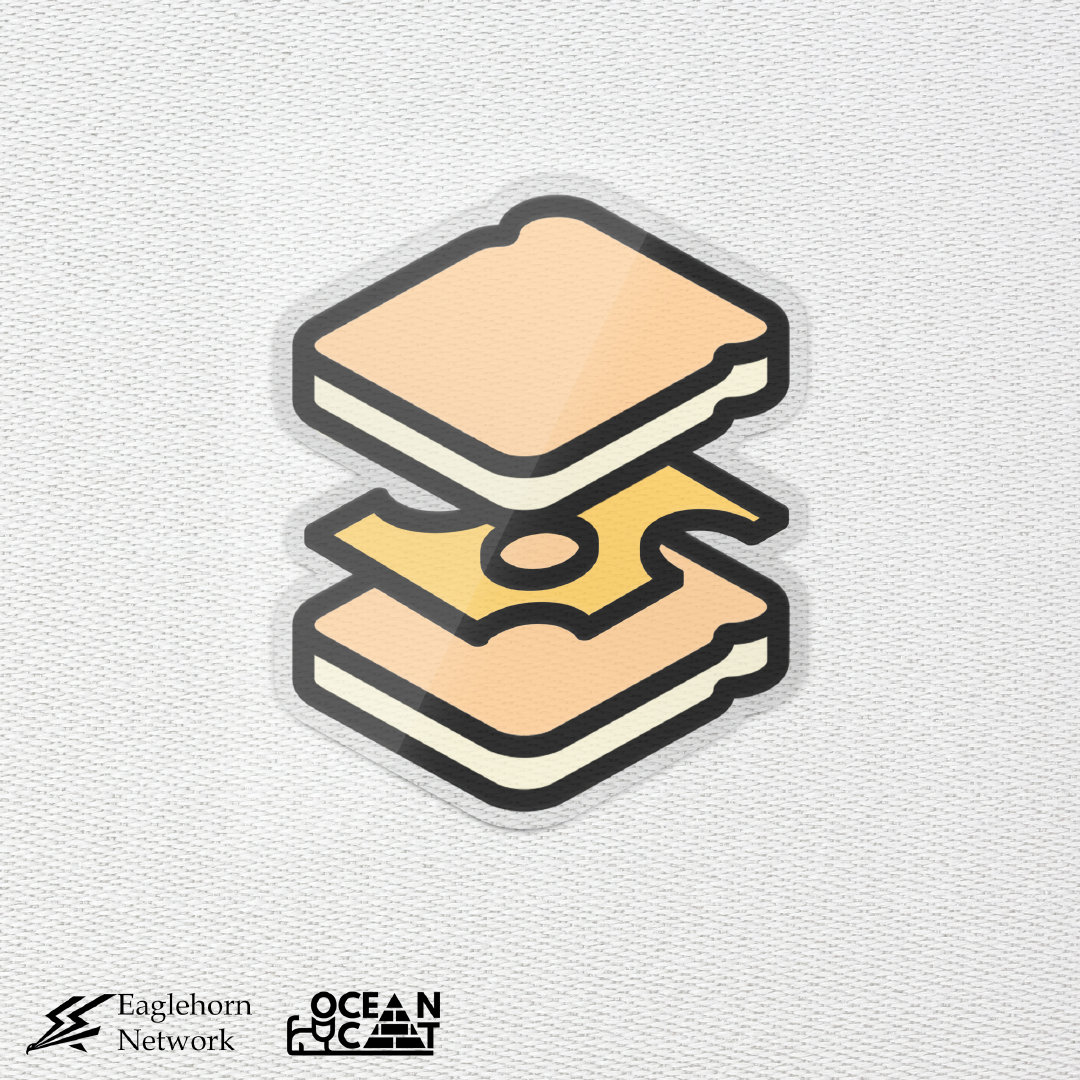

Supplement: Supplementary file 9 — Supplementary Software 1 [file 41467_2023_41859_MOESM9_ESM.zip › Supplementary Software/AutoTransfer/gui/bread_and_cheese.jpg]

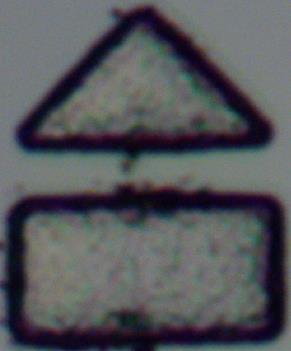

Supplement: Supplementary file 9 — Supplementary Software 1 [file 41467_2023_41859_MOESM9_ESM.zip › Supplementary Software/AutoTransfer/vision/templates/1.jpg]

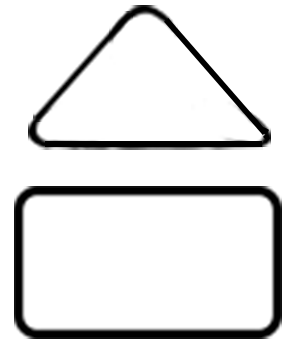

Supplement: Supplementary file 9 — Supplementary Software 1 [file 41467_2023_41859_MOESM9_ESM.zip › Supplementary Software/AutoTransfer/vision/templates/1.png]

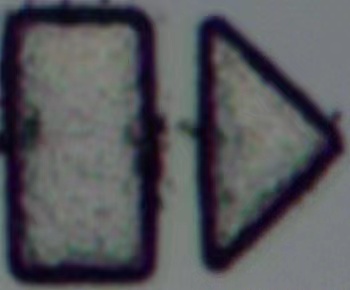

Supplement: Supplementary file 9 — Supplementary Software 1 [file 41467_2023_41859_MOESM9_ESM.zip › Supplementary Software/AutoTransfer/vision/templates/2.jpg]

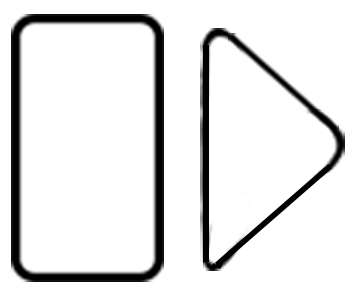

Supplement: Supplementary file 9 — Supplementary Software 1 [file 41467_2023_41859_MOESM9_ESM.zip › Supplementary Software/AutoTransfer/vision/templates/2.png]

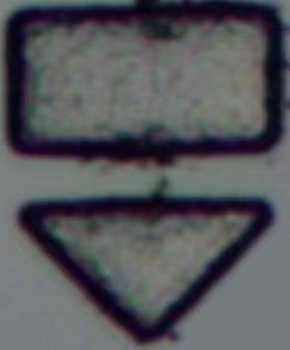

Supplement: Supplementary file 9 — Supplementary Software 1 [file 41467_2023_41859_MOESM9_ESM.zip › Supplementary Software/AutoTransfer/vision/templates/3.jpg]

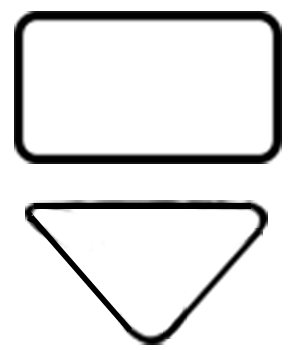

Supplement: Supplementary file 9 — Supplementary Software 1 [file 41467_2023_41859_MOESM9_ESM.zip › Supplementary Software/AutoTransfer/vision/templates/3.png]

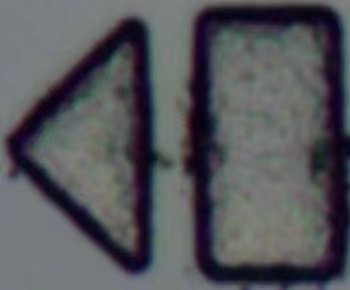

Supplement: Supplementary file 9 — Supplementary Software 1 [file 41467_2023_41859_MOESM9_ESM.zip › Supplementary Software/AutoTransfer/vision/templates/4.jpg]

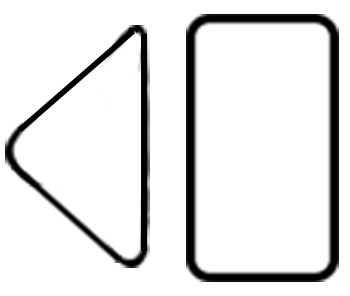

Supplement: Supplementary file 9 — Supplementary Software 1 [file 41467_2023_41859_MOESM9_ESM.zip › Supplementary Software/AutoTransfer/vision/templates/4.png]

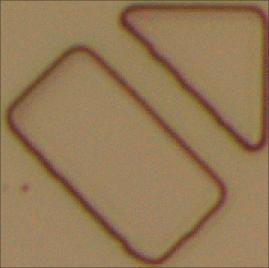

Supplement: Supplementary file 9 — Supplementary Software 1 [file 41467_2023_41859_MOESM9_ESM.zip › Supplementary Software/AutoTransfer/vision/templates/5.jpg]

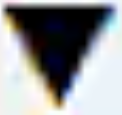

Supplement: Supplementary file 9 — Supplementary Software 1 [file 41467_2023_41859_MOESM9_ESM.zip › Supplementary Software/AutoTransfer/vision/templates/down.png]

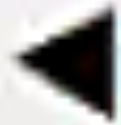

Supplement: Supplementary file 9 — Supplementary Software 1 [file 41467_2023_41859_MOESM9_ESM.zip › Supplementary Software/AutoTransfer/vision/templates/left.png]

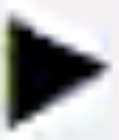

Supplement: Supplementary file 9 — Supplementary Software 1 [file 41467_2023_41859_MOESM9_ESM.zip › Supplementary Software/AutoTransfer/vision/templates/right.png]

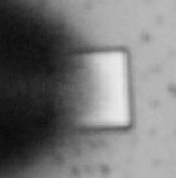

Supplement: Supplementary file 9 — Supplementary Software 1 [file 41467_2023_41859_MOESM9_ESM.zip › Supplementary Software/AutoTransfer/vision/templates/tip_mask_light_2.png]

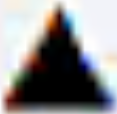

Supplement: Supplementary file 9 — Supplementary Software 1 [file 41467_2023_41859_MOESM9_ESM.zip › Supplementary Software/AutoTransfer/vision/templates/up.png]

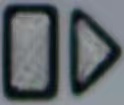

Supplement: Supplementary file 9 — Supplementary Software 1 [file 41467_2023_41859_MOESM9_ESM.zip › Supplementary Software/AutoTransfer/vision/templates/╨┬╜¿╬─╝■╝╨/1.jpg]

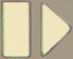

Supplement: Supplementary file 9 — Supplementary Software 1 [file 41467_2023_41859_MOESM9_ESM.zip › Supplementary Software/AutoTransfer/vision/templates/╨┬╜¿╬─╝■╝╨/1.png]

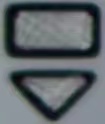

Supplement: Supplementary file 9 — Supplementary Software 1 [file 41467_2023_41859_MOESM9_ESM.zip › Supplementary Software/AutoTransfer/vision/templates/╨┬╜¿╬─╝■╝╨/2.jpg]

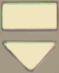

Supplement: Supplementary file 9 — Supplementary Software 1 [file 41467_2023_41859_MOESM9_ESM.zip › Supplementary Software/AutoTransfer/vision/templates/╨┬╜¿╬─╝■╝╨/2.png]

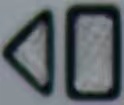

Supplement: Supplementary file 9 — Supplementary Software 1 [file 41467_2023_41859_MOESM9_ESM.zip › Supplementary Software/AutoTransfer/vision/templates/╨┬╜¿╬─╝■╝╨/3.jpg]

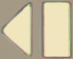

Supplement: Supplementary file 9 — Supplementary Software 1 [file 41467_2023_41859_MOESM9_ESM.zip › Supplementary Software/AutoTransfer/vision/templates/╨┬╜¿╬─╝■╝╨/3.png]

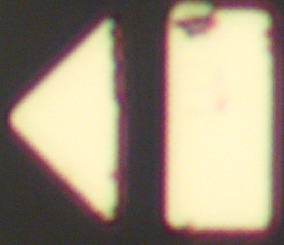

Supplement: Supplementary file 9 — Supplementary Software 1 [file 41467_2023_41859_MOESM9_ESM.zip › Supplementary Software/AutoTransfer/vision/templates/╨┬╜¿╬─╝■╝╨/30.png]

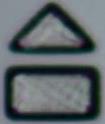

Supplement: Supplementary file 9 — Supplementary Software 1 [file 41467_2023_41859_MOESM9_ESM.zip › Supplementary Software/AutoTransfer/vision/templates/╨┬╜¿╬─╝■╝╨/6.jpg]

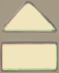

Supplement: Supplementary file 9 — Supplementary Software 1 [file 41467_2023_41859_MOESM9_ESM.zip › Supplementary Software/AutoTransfer/vision/templates/╨┬╜¿╬─╝■╝╨/l4.png]
